# Clinical Questions Asked by Long-Term Care Providers Through eConsult: A Retrospective Study

**DOI:** 10.1177/23337214211032055

**Published:** 2021-08-28

**Authors:** Celeste Fung, Soha Shah, Mary Helmer-Smith, Cheryl Levi, Erin Keely, Clare Liddy

**Affiliations:** 1 St. Patrick’s Home of Ottawa, Ottawa, ON, Canada; 2 Department of Family Medicine, 6363 University of Ottawa, Ottawa, ON, Canada; 3 Ontario eConsult Centre of Excellence, 27337 The Ottawa Hospital, Ottawa, ON, Canada; 4 C.T. Lamont Primary Health Care Research Centre, 152971 Bruyère Research Institute, Ottawa, ON, Canada; 5 Emergency Department Outreach Program, 27337 The Ottawa Hospital, Ottawa, ON, Canada; 6 Department of Medicine, 6363 University of Ottawa, Ottawa, ON, Canada; 7 Division of Endocrinology/Metabolism, 27337 The Ottawa Hospital, Ottawa, ON, Canada

**Keywords:** long-term care, geriatrics, telemedicine, specialist care

## Abstract

**Introduction:**

eConsult allows primary care providers (PCPs) to access timely specialist advice and informs patient care. To understand the use of eConsult in long-term care (LTC) settings, we examined the clinical content and types of questions asked by LTC PCPs.

**Methods:**

A descriptive, retrospective study of eConsults submitted through the Champlain BASE™ eConsult Service between January 1, 2017, and December 31, 2018, by LTC PCPs was conducted. Cases were classified using validated taxonomies. Descriptive statistics were generated for content and question type classifications, service utilization data, and close-out survey responses.

**Results:**

22 LTC PCPs submitted 113 eConsults. They sought advice about drug treatment (58%), diagnosis (44%), and management (38%) in a breadth of clinical areas, often skin-related (39%). Long-term care PCPs frequently asked more than one question type (42%). They received advice within 1 week (91%) and rated eConsult as very helpful and educational. Three case examples are presented.

**Conclusion:**

This study demonstrates the type of advice LTC PCPs are seeking through eConsult and its usefulness in this setting. Long-term care stakeholders are encouraged to consider implementing eConsult in other regions, as a means to improve access to timely specialist advice, support clinical decision-making, and improve residents’ quality of life.

## Introduction

There is a growing population of residents living in long-term care (LTC) who have multiple chronic illnesses, increased medical complexity, and numerous comorbidities (Ng et al., 2020; Ontario Long Term Care Association [OLTCA], 2019). Between 2011–2012 and 2017–2018, LTC residents experienced an increased prevalence of common diseases, such as heart/circulation diseases (7.8%; 76.3% total), hypertension (4.5%; 64.5% total), dementia (4.0%; 63.9% total), and gastrointestinal diseases (6.8%; 26.2% total) (Canadian Institute for Health Information [CIHI], 2012, 2018). More than half of LTC residents are age 85 years and older, and nearly two-thirds of residents take 10 or more prescription medications (CIHI, 2014; OLTCA, 2019). As a result, physicians and nurse practitioners (hereafter referred to collectively as primary care providers (PCPs)) manage increasingly complex care in LTC homes.

Advice from colleagues and specialists can help to inform clinical decision-making by PCPs in LTC, but access to specialists within LTC homes is limited. In Ontario, the rate of specialist visits outside of the LTC home is much higher than within the home, as residents are most often required to attend external appointments to receive specialist advice (Shaver et al., 2020). These appointments are challenging for most residents due to the barriers associated with mobility and transfers, transportation, and coordinating caregiver escorts. Thus, referrals may not be initiated (Helmer-Smith et al., 2020) and conditions may go untreated or managed without the advice of specialists. In the case of a persistent skin condition, for example, this can have a significant impact on the resident’s quality of life (Shah & Coates, 2006). During the COVID-19 pandemic, there has been particular attention focused on avoiding external transfers and minimizing interactions with medical personnel to reduce the risk of exposure and the potential for institutional outbreaks. These measures, however necessary, likely further reduce access to specialists.

A growing number of digital health tools are available to support PCPs’ access to specialist advice. These include synchronous (real-time) and asynchronous tools, which facilitate PCP-to-specialist communication, either with or without the resident present. Though synchronous tools such as video-visits and telephone-based services have proven beneficial in this setting (Canadian Agency for Drugs and Technologies in Health, 2015; Edirippulige et al., 2013), these services often require advanced technology, coordination of provider schedules, and staff support. The initial investment and workflow/process challenges associated with interactive audio- and video-facilitated consults are barriers to adoption (Driessen et al., 2018). In contrast, asynchronous communication between PCPs and specialists, such as through electronic consultation (eConsult), allows PCPs to submit clinical questions directly to specialists and for specialists to respond on their own time, thereby eliminating the need to coordinate provider schedules and for high-bandwidth connections. In community settings, eConsult services improve timely access to specialist advice, are effective clinical-decision support tools, improve patient experience, have high provider satisfaction, build PCP-specialist relationships, are sustainable, and are cost-effective (Barnett et al., 2017; Lee et al., 2018; Liddy et al., 2016, 2019; Liddy & Keely, 2018; Vimalananda et al., 2015). Though the feasibility of using eConsult in LTC was recently demonstrated (Helmer-Smith et al., 2020), little is known about how PCPs are using it in this setting or the type of advice they are seeking.

No previous studies have described the types of questions posed by LTC PCPs to specialists, either through referral forms or eConsult services. Taxonomy studies have been conducted in primary care settings and specialty clinics to characterize the information and learning needs of providers, inform continuing medical education, and understand how eConsult is used by clinicians working in primary care (Archibald, Liddy, et al., 2018; Bjerre et al., 2013; Del Fiol, Weber, et al., 2014; Del Fiol, Workman, et al., 2014; Ely et al., 2000). Using a similar approach, this study will describe the clinical topics and types of questions asked through eConsult by physicians and nurse practitioners working in LTC and demonstrate its usefulness to LTC clinicians.

## Methods

### Study Design

We conducted a descriptive, retrospective study of all eConsult cases submitted to the Champlain BASE™ eConsult Service between January 1, 2017, and December 31, 2018, by PCPs working in LTC.

### Setting

The Champlain BASE™ eConsult service is based in a health region in Eastern Ontario with a population of 1.3 million people. The region includes a mostly rural geography with a single large urban center. It is home to 58 of Ontario’s 630 LTC homes. Though some LTC homes in Ontario have full-time PCPs, the average LTC physician practices in more than one LTC home on a part-time basis in addition to their community-based practice (Lam et al., 2012). It is more common for nurse practitioners to work full-time in a single LTC home (63%); however, many provide care in several homes (Ontario Ministry of Long-Term Care, 2020). They are primarily staffed through government allocated positions; directly by LTC homes; or through hospital programs, such as Nurse-Led Outreach Teams (Nurse Practitioners’ Association of Ontario, 2020).

### Intervention: Champlain BASE™ eConsult Service

The Champlain BASE™ (Building Access to Specialists through eConsultation) eConsult Service, established in 2010, is a secure web-based tool that allows a PCP (physician or nurse practitioner) timely access to specialist advice for all patients and residents, often eliminating the need for an in-person specialist visit. eConsult offers asynchronous, direct provider-to-specialist communication. Simply accessed through the online portal, eConsults can be submitted by PCPs from practically any location at any time. To submit an eConsult, PCPs enter their question and relevant case details into a short form, select a specialty group to send the question to, and optionally attach any pertinent documents (e.g., bloodwork or images). The case is assigned to a specialist who responds within 1 week. The service allows for iterative communication between the PCP and specialist, similar to email communication, until the case is closed. Upon closing the case, PCPs complete a mandatory five-question close-out survey (Table 1). Specialists are remunerated for time spent responding to the question. Remuneration is prorated at $200/hour and based on their self-reported billing time. Program funding, including remuneration, is provided by the Ontario Ministry of Health.

**Table 1.** Champlain BASE™ eConsult primary care provider close-out survey questions.

Q1. Which of the following best describes the outcome of this eConsult for your patient: 1. I was able to confirm a course of action that I originally had in mind 2. I got good advice for a new or additional course of action that I will be implementing 3. I got good advice for a new or additional course of action that I am not able to implement 4. None of the above (please comment)
Q2. As a result of this eConsult, would you say that: 1. Referral was originally contemplated but now avoided at this stage 2. Referral was originally contemplated and is still needed 3. Referral was not originally contemplated and is still not needed 4. Referral was not originally contemplated, but eConsult resulted in a referral being initiated 5. Other (please explain)
Q3. How helpful and/or educational was this response in guiding your ongoing evaluation or management of the patient? (Minimal) 1 2 3 4 5 (Very Valuable)
Q4. This eConsult addresses an important clinical problem that should be incorporated into upcoming CME events. (Strongly Disagree) 1 2 3 4 5 (Strongly Agree)
Q5. We would value any additional feedback you provide (Comments for the specialist will be forwarded to her/him): (Free text comment box)

eConsult = electronic consultation.

### Case Identification

Primary care providers were identified as working in LTC by cross-referencing the organization listed in their eConsult account with a list of LTC homes in Ontario. All cases submitted during the study period by identified LTC PCPs were included for analysis.

### Data Collection

We used routine service utilization data. Case-level data include the type of specialty requested, type of referring PCP (physician or nurse practitioner), specialist response time, and a log of all exchanges between the providers. Close-out survey response data were included for analysis. No identifying patient data were included for analysis, and patients did not participate directly in the study. Thus, patient consent was not sought and could not be obtained.

### Data Analysis

Following established methodology (Archibald, Liddy, et al., 2018; Archibald, Stratton, et al., 2018; Bjerre et al., 2013; Keely et al., 2018), case transcripts were reviewed by a team of LTC PCPs consisting of a LTC Medical Director, Nurse Practitioner, and Care of the Elderly Medical Resident (CF, CLe, SS). Reviewers classified cases retrospectively using modified versions of two existing and validated taxonomies: the International Classification for Primary Care, version 2 (ICPC-2) (WONCA International Classification Committee, 2005) and Ely et al. (2000) taxonomy of generic clinical questions (TGCQ). The ICPC-2 was used for content classification (i.e., clinical topic). The TGCQ was used for question type classification. These were modified by reviewers 1 and 2 to include clinical topic and question type areas relevant to LTC. The final taxonomies include content categories such as cardiovascular, urological, and neurological and question types such as diagnostic, drug treatment, and management. The complete lists are in the Supplementary Material.

The first 40 cases were classified independently by each reviewer and compared to validate the study taxonomy and ensure inter-rater reliability. During this stage, we modified the taxonomy to allow for a second question type to be classified. If a case included two questions that fell within one category (e.g., drug prescription), a single question type was classified. If a case included two clear questions about both diagnosis and treatment, for example, the second question type option was used to capture this. Consensus was reached on any case classifications where there was variance by re-reviewing and discussing the case transcript. The remaining cases were divided amongst reviewers, with each classified by a single reviewer.

Descriptive analysis was used to examine content and question type classifications, service utilization data, and responses to the mandatory close-out survey. Summary statistics, primarily measures of frequency, were generated and are presented.

### Case Examples

Case examples are commonly used for case-based learning in medical education, as they allow learners to connect theory to practice (McLean, 2016; Thistlethwaite et al., 2012). Three LTC eConsult case examples were purposively selected by the LTC PCP reviewer team to aid readers in understanding the type of information exchanged, demonstrate the breadth of specialist advice that can be obtained through eConsult, and illustrate the usefulness of this tool in the LTC setting. All patient and provider identifying information (e.g., resident sex and age) was omitted or modified to ensure anonymity.

## Results

113 eConsult cases were submitted between January 1, 2017, and December 31, 2018, by 22 PCPs working in LTC and included for classification. 49% of cases were submitted by nurse practitioners. 91% of cases received a response from a specialist in 1 week or less.

Long-term care PCPs most frequently asked skin-related questions, followed by those about cardiovascular and musculoskeletal issues (Table 2).

**Table 2.** Classification of LTC electronic consultation cases by clinical topic.

Clinical Topic	Number of eConsults	Percentage of eConsults (%)
Skin	44	39
Cardiovascular	9	8
Musculoskeletal	9	8
Neurological	8	7
Digestive	6	5
Pain	6	5
Urological	5	4
Abnormal result or investigation NOS	3	3
Adverse effect	3	3
Blood, blood forming organs, and immune mechanism	3	3
Endocrine/metabolic and nutritional	3	3
Infection NOS	3	3
Respiratory	3	3
Ear	2	2
Female genital	2	2
Eye	1	1
Male genital	1	1
Malignancy NOS	1	1
Psychological	1	1

NOS = not otherwise specified; LTC = long-term care; eConsult = electronic consultation.

**Figure 1.**
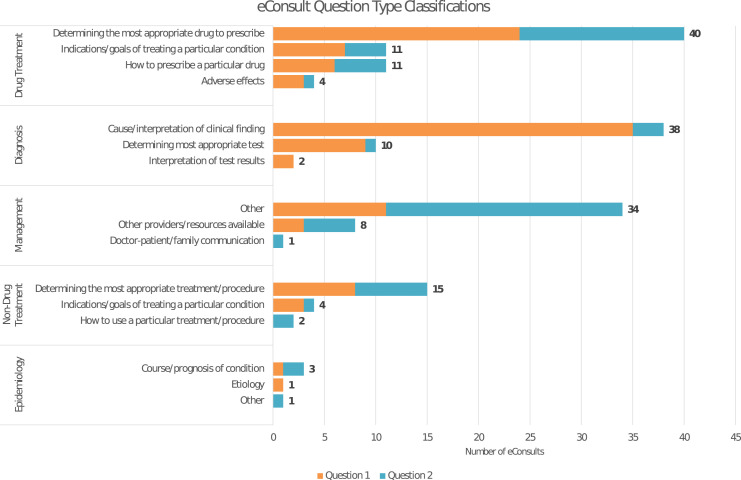
Classification of long-term care electronic consultation cases by question type.

In 42% of cases, LTC PCPs asked more than one question type. The most common questions pertained to drug treatment (58%, 66/113), diagnosis (44%, 50/113), and management (38%, 43/113). Generally, when more than one question type was asked, the LTC PCPs first asked about the cause/interpretation of a clinical finding and then how best to manage care or determine the most appropriate drug to prescribe (Figure 1).

Despite the frequency of cases with more than one question type, 79% of cases were closed after a single question/response interaction. The remaining cases involved continued communication/follow-up through the eConsult portal between the PCP and specialist.

66 drug treatment question types were classified from 56 of the 113 cases. The majority of these cases were about skin (34%, 19/56), neurology (13%, 7/56), and pain (9%, 5/56).

When asked how helpful and/or educational the specialist’s response was in guiding ongoing evaluation or management of the resident’s care, 86% of LTC PCPs rated the response as 4/5 or 5/5 in value, with a mean rating of 4.3. Additionally, 53% of PCPs either agreed or strongly agreed their eConsult addressed an important clinical problem that should be incorporated into upcoming continuing medical education events.

### Case Examples

#### Case example 1: Diagnosis and drug treatment of a skin condition

The resident has a history of dementia, skin conditions, and other chronic conditions. They are wheelchair bound and require lift transfers. The PCP sent an eConsult to dermatology, relaying the resident has a skin lesion located centrally on the low-back which is progressively accumulating scaly-type keratotic material over the last 4 months. The PCP provided a picture and detailed description of the lesion, suggesting Bowen’s as a possibility. They noted “this patient is very frail and arranging transfer for further assessment or procedure would be quite challenging. If any in-home treatments could be considered as a first step, the patient and family would surely appreciate this option.”

The dermatologist responded the next day stating the raised portion of the lesion has a “cutaneous horn” appearance and that they agree that Bowen’s Disease (Squamous Cell Carcinoma in-situ) is a possibility. The specialist noted that a skin biopsy would provide a histological diagnosis, but they suggested a topical cream treatment regimen, if biopsy is problematic.

#### Case example 2: Medication adverse effects and deprescribing

The resident had resided in LTC for over 4 years. The PCP described their history, which includes dementia with resistance to care, depression, ADHD, and a remote overdose requiring hospitalization. The PCP provided a detailed medication history, including history of deprescribing, and recent MMSE assessment values. They described the resident is fixed in their routine, and staff report no behavioral concerns; however, staff report a gradual deterioration in gait with increased unsteadiness. The PCP expressed concern that the resident is at increased risk for falls, noting specific observations, and wrote “suggestions and recommendations for medication adjustment to reduce risk for falls would be very much appreciated including an approach to taper psychotropic medications if no signs or symptoms are observed of recurring depression or worsening behaviours.”

In their response 3 hours later, the geriatric medicine specialist applauded the PCPs deprescribing actions thus far and suggested trialing further reduction of a medication dose for 7 days, while monitoring for mood/behavior changes. They also suggested the PCP involve Geriatric Psychiatry to help with behavior management and identified a specific provincial support team as a resource. The geriatrician indicated that, if the dementia is vascular, the PCP may be observing progression of the disease and not medication side-effects but that decrease or discontinuation of medications that are no longer effective would be beneficial in both cases. The geriatrician closed with “Please do not hesitate to re-consult if needed.”

#### Case example 3: Urological management and non-drug treatment

The PCP stated the reason for consultation was frequent foley catheter changes resulting from catheter occlusions. They described a specific resident’s medical history and details of catheter changes. The PCP explained they had searched the literature for prevention strategies and listed some identified strategies. The PCP asked the urologist what they would recommend to reduce or prevent these frequent urinary catheter blockages, suggesting frequent irrigation, and thanked them.

The next day, the urologist asked follow-up questions and provided lengthy and detailed information about the principles of long-term indwelling catheter management, particularly regarding catheter size, catheter material, and the controlling factors that contribute to clogging of the catheter (e.g., fluid intake, presence of gross hematuria, and recurrent urinary tract infections). They agreed regular catheter flushing could help to prevent clogging, but explained it tends not to be as critical if the catheter is of good size and gross hematuria and UTIs are limited. The urologist gave additional information regarding irrigation solutions, how to encourage passive drainage, and complications that can arise from the specific indication for use. They provided seven specific recommendations, regarding catheter size, catheter material, catheter elevation, fluid intake, frequency of changes, minimization of gross hematuria, and foley flushing.

Three days later, the PCP responded with answers to all the urologist’s questions, provided information regarding implementation of the recommendations, and made additional inquiries. For example, the resident was using the catheter size recommended, so the PCP asked if they should go up a size. The PCP thanked the urologist for their recommendations.

One hour later, the urologist agreed with the PCP’s suggestion to increase catheter size and recommended trying the increased size prior to changing catheter material. They confirmed that the rest of their recommendations would stay the same, if applicable to the resident.

## Discussion

Our study demonstrates that eConsult has been used effectively by LTC physicians and nurse practitioners to seek specialist advice about treatment, diagnosis, and management of residents’ care regarding various clinical topics, most commonly skin-related issues. Complex, resident-specific questions were addressed in a timely and satisfactory way, and PCPs indicated they received a helpful and educational response back from the specialist. The case examples provided exemplify the quality and helpfulness of the specialist’s response to the PCPs.

The high proportion of skin-related cases (39%) is not surprising, given dermatology is the most commonly accessed specialty group overall on the Champlain BASE™ eConsult Service (20% of cases) and the prevalence of dermatologic issues in LTC settings (Hahnel et al., 2017). A study evaluating the clinical questions raised by providers caring for older adults in geriatric and community clinics found that most questions were about drug treatment (33%) and diagnosis (13%) (Del Fiol, Weber, et al., 2014). Through eConsult, LTC PCPs also most frequently ask drug treatment and diagnostic questions; however, in this setting they have a much higher rate of questions about care management than reported in geriatric and community clinics (Del Fiol, Weber, et al., 2014). Nurse practitioners submit a greater proportion of the cases from LTC (49%), in comparison to cases from the service overall (11%). To explain this, it could be informative to compare the ratio of nurse practitioners to physicians in LTC and other settings, but the exact number of nurse practitioners working in LTC is not clear, given the varied funding structures and lack of a central database (Nurse Practitioners’ Association of Ontario, 2020; Ontario Ministry of Long-Term Care, 2020). This utilization by nurse practitioners highlights the importance of offering eConsult to both physicians and nurse practitioners, particularly for nurse practitioners who may lack a physician network and consequently have greater challenges in accessing specialist advice. The helpfulness and educational value of eConsult reported in this study is consistent with trends for the overall service and results from previous research. 91% of PCPs rate the response as helpful and 60% of PCPs agree the case would be educational for their peers (Champlain BASE™ eConsult Service, 2017–2018). Further, previous work found that eConsult provided LTC PCPs with a new or additional course of action in 60% of cases (Helmer-Smith et al., 2020).

Given the limited access to specialist advice within LTC homes, challenges associated with external transfer, restrictions related to COVID-19 and infection control, and eConsult’s proven ability to facilitate high-quality, specific and helpful PCP-specialist communication about a residents’ care, eConsult is highly applicable to the LTC setting. Through eConsult, residents and their PCPs are able to obtain specialist advice to treat/manage the residents’ condition(s) and inform care planning, often without the need for a face-to-face specialist visit (70%) (Helmer-Smith et al., 2020). In this way, eConsult is improving equitable access to specialist advice, supporting clinical decision-making, helping to address traditionally unmet needs, and improving residents’ quality of life. Clinicians, policymakers, and resident/family advocates are encouraged to consider these findings and the potential impact of eConsult for LTC residents and providers if it were to be adopted more broadly.

This study is the first of its kind to explore the clinical questions asked through eConsult by PCPs working in LTC. It provides a foundation for further research into the information and learning needs of LTC PCPs, which could inform continuing medical education and support capacity building in the sector. Future studies could explore whether there are differences in questions submitted, and thus learning needs, between nurse practitioners and physicians and across geographic regions. Evaluation of the adoption, use, and impact of eConsult in LTC settings will continue as implementation of eConsult continues to expand across the province through the Ontario eServices Program.

Our study has several limitations. A convenience sample, with a small sample size, from a single health region in Ontario was used. Therefore, the results may not be generalizable to other regions and jurisdictions. Additionally, all cases initiated by a PCP with a LTC home identified as their primary organization in their eConsult account were included in this study. This method of case identification may have resulted in the inclusion of cases submitted on behalf of persons living in the community seen by the PCP outside of the LTC setting. It may also have resulted in exclusion of cases submitted on behalf of LTC residents by PCPs whose primary organization is a community clinic, not the LTC home. Despite these limitations, use of an established methodology and validated clinical taxonomy tools (the TGCQ and ICPC-2) strengthen this study and allow for comparison to other research that have followed a similar approach.

## Conclusion

This study demonstrates the type of advice LTC PCPs are asking for through eConsult and its usefulness in this setting. Primary care providers working in LTC use eConsult to seek diagnostic, treatment, and management advice from specialists in most clinical areas, particularly regarding skin-related issues. Use of eConsult has permitted these PCPs to obtain timely, resident-specific, and practical recommendations to optimize care for the complex and vulnerable LTC resident population, without the need for external transfer or in-person specialist visits. This is of particular value during the COVID-19 pandemic. Long-term care stakeholders are encouraged to consider implementing eConsult in other LTC homes and health regions, as a means to improve access to timely specialist advice, support clinical decision-making, and improve residents’ quality of life.

## Supplemental Material

sj-pdf-1-ggm-10.1177_23337214211032055 – Supplemental Material for Clinical Questions Asked by Long-Term Care Providers Through eConsult: A Retrospective Study Click here for additional data file. Supplemental Material, sj-pdf-1-ggm-10.1177_23337214211032055 for Clinical Questions Asked by Long-Term Care Providers Through eConsult: A Retrospective Study by Celeste Fung, Soha Shah, Mary Helmer-Smith, Cheryl Levi, Erin Keely and Clare Liddy in Gerontology and Geriatric Medicine
